# Influence of 3D Printing Parameters on the Mechanical Stability of PCL Scaffolds and the Proliferation Behavior of Bone Cells

**DOI:** 10.3390/ma15062091

**Published:** 2022-03-11

**Authors:** Fabian Huber, David Vollmer, Johannes Vinke, Bianca Riedel, Sergej Zankovic, Hagen Schmal, Michael Seidenstuecker

**Affiliations:** 1 G.E.R.N. Center of Tissue Replacement, Regeneration & Neogenesis, Department of Orthopedics and Trauma Surgery, Medical Center—Albert-Ludwigs-University of Freiburg, Faculty of Medicine, Albert-Ludwigs-University of Freiburg, Hugstetter Straße 55, 79106 Freiburg, Germany; fabian.huber@uniklinik-freiburg.de (F.H.); david.vollmer@uniklinik-freiburg.de (D.V.); bianca.riedel@uniklinik-freiburg.de (B.R.); sergej.zankovic@uniklinik-freiburg.de (S.Z.); hagen.schmal@uniklinik-freiburg.de (H.S.); 2 Institute for Applied Biomechanics, Offenburg University, Badstraße 24, 77652 Offenburg, Germany; vinke@hs-offenburg.de

**Keywords:** 3D printing, PCL scaffold, geometry variation, mechanical properties, biocompatibility

## Abstract

Introduction The use of scaffolds in tissue engineering is becoming increasingly important as solutions need to be found for the problem of preserving human tissue, such as bone or cartilage. In this work, scaffolds were printed from the biomaterial known as polycaprolactone (PCL) on a 3D Bioplotter. Both the external and internal geometry were varied to investigate their influence on mechanical stability and biocompatibility. Materials and Methods: An Envisiontec 3D Bioplotter was used to fabricate the scaffolds. First, square scaffolds were printed with variations in the strand width and strand spacing. Then, the filling structure was varied: either lines, waves, and honeycombs were used. This was followed by variation in the outer shape, produced as either a square, hexagon, octagon, or circle. Finally, the internal and external geometry was varied. To improve interaction with the cells, the printed PCL scaffolds were coated with type-I collagen. MG-63 cells were then cultured on the scaffolds and various tests were performed to investigate the biocompatibility of the scaffolds. Results: With increasing strand thickness and strand spacing, the compressive strengths decreased from 86.18 + 2.34 MPa (200 µm) to 46.38 + 0.52 MPa (600 µm). The circle was the outer shape with the highest compressive strength of 76.07 + 1.49 MPa, compared to the octagon, which had the lowest value of 52.96 ± 0.98 MPa. Varying the external shape (toward roundness) geometry, as well as the filling configuration, resulted in the highest values of compressive strength for the round specimens with honeycomb filling, which had a value of 91.4 + 1.4 MPa. In the biocompatibility tests, the round specimens with honeycomb filling also showed the highest cell count per mm^2^, with 1591 ± 239 live cells/mm^2^ after 10 days and the highest value in cell proliferation, but with minimal cytotoxic effects (9.19 ± 2.47% after 3 days).

## 1. Introduction

The German health care system will need to be assessed and reconsidered in the future in the face of demographic change, as the number of illnesses such as diabetes, high blood pressure, or stroke will increase in the coming years. One reason for this is that the ratio between young and old people continues to shift and society as a whole is “getting older” [1,2]. The increased incidence of bone defects and injuries of any kind is thus becoming more and more important [3,4]. This is also reflected in the data of the Federal Statistical Office. Every second person in Germany is over 45 years old and every fifth over the age of 66 [5]. The same pattern applies to the European Union (EU), where every fifth person is over 65 [2]. In 2019, a total of 17,229,013 surgeries were performed, with implantation of an endoprosthesis in the hip being the sixth most common surgical procedure, with 243,477 procedures in Germany. The implantation of endoprostheses in the knee also played a significant role, with 193,759 procedures in Germany alone [6]. Currently, the repair of bone defects is mainly accomplished with implants made of metal alloys. These metal alloys show good properties, both in strength and toughness. However, the problem of stress shielding [7] should be mentioned; this results from the mismatch of the elastic modulus (Young’s modulus) between metals (100 GPa to 250 GPa) and human bone (7 GPa to 25 GPa) [8,9]. The higher stiffness of metal implants ensures that they take on a significant portion of the stress [10]. This stress shielding of the bone decreases the bone density and the bone cannot heal optimally, which can lead to a new fracture [11]. In addition, the procedure requires a second surgery, in which the additional metallic materials (e.g., metal plates to fix the bone defect) are removed after the bone heals because they are not biodegradable. For these reasons, efforts have already been made to either adapt the mechanical properties of the metallic implants to those of the bone by investigating other alloys [12,13,14] or, for example, by foaming the material [15]. Efforts are also underway to replace the metallic materials entirely with other materials, such as polymeric composites [16] or carbon-fiber-reinforced composites [17,18]. Thus, the search for biodegradable material has the greatest relevance, so that a second surgical intervention can be avoided and the material will degrade in the body after a certain time [11]. Another concept for repairing bone defects is autogenous bone grafting [19]. In this approach, the defective bone is replaced with a healthy bone from elsewhere in the patient’s own body. Additionally, allogeneic bone grafting would be an option. Here, the bone substitute is used from another person [20]. However, both treatment methods have disadvantages. Autogenous bone grafting may result in wound infection, increased blood loss, or donor site morbidity [21]. Infections transmitted to the patient by the donor and the risk of rejection of the bone implant are obstacles to successful allogeneic bone grafting [22]. Due to this problem, biodegradable materials are increasingly used, with polyglycolic acid [23], polylactic acid [24], and polycaprolactone (PCL) [25] being the most common. Due to their chemical analogy to the inorganic components of bone, bioceramics are equally well suited [26]. Magnesium, which is a biodegradable metal, also has great potential, offering interesting mechanical properties [27]. Finally, preventing the release of toxic substances to avoid the functional impairment of biological tissue is an important aspect [26]. The applications of these materials are feasible with modern techniques, such as tissue engineering (TE) or even using a 3D printer. Thus, bone-substitute scaffolds (scaffolds) can be fabricated from biodegradable materials, but they must be designed to ensure an optimal environment for the formation of new bone [26]. In addition to the properties described above, the scaffolds must also have an optimal geometry. Thus, for osteogenesis, the scaffolds should represent a copy of the bone-like structure, function, and morphology. Human bone is formed mainly from the material hydroxyapatite, and this is predominantly deposited in type-I collagen, which is an organic matrix [28]. Furthermore, bone can be macroscopically divided into the substantia corticalis and substantia spongiosa. The substantia corticalis is a solid material and is mainly found on the bone’s surface. Finally, inside the bone, one encounters the substantia spongiosa, which represents a spongy meshwork [29]. Due to this spongy meshwork, the bone interior exhibits a porosity of 50 to 90% and has a pore size of approximately one millimeter in diameter. In addition, four cell types are found exclusively in bone tissue: the osteoblasts, osteoclasts, osteocytes, and bone lining cells [28]. Thus, the crucial factors for bone wound healing are mainly the pore size and the interconnectivity of the porous structure. Various studies also indicate that the pore shape and surface curvature can have significant effects on the generation process of bone [30]. Sung Wook Choi et al. [31] have attempted to fabricate scaffolds with uniform pore sizes, thus eliciting the optimal pore size for cell attachment behavior. It was found that different pore sizes show an influence on the growth behavior of cells. Karageorgiou et al. [28] further reported that the smallest possible pore size of a scaffold to achieve the positive regeneration of mineralized bone can be around 100 µm. In particular, pore sizes in the range of 150 µm to 200 µm are suitable as favorable sizes for good bone ingrowth. However, another study was able to show that bone growth could also be observed in scaffolds with pore sizes of between 300 µm and 800 µm, and no significant differences were determined for the different pore sizes [32]. So far, there have been very few studies (we know of only one, and it was based on solid scaffolds without pores [33]) on the effects of the geometry of the printed scaffolds on mechanical strength, as well as the influence of the geometry on cell growth. The aim of the present work was to determine the influence of the geometry of 3D-printed polycaprolactone (PCL) scaffolds on the mechanical strength and proliferation of bone cells. PCL was chosen as an example because it is very good for 3D printing, there are already many publications about it, and we can compare it with previous work.

## 2. Materials and Methods

### 2.1. Materials

PCL, average Mn 45.000, and N-(3-Dimethylaminopropyl)-N′-ethyl carbodiimide hydrochloride (EDC) and fetal bovine serum (FBS) were purchased from Sigma Aldrich (Merck, Darmstadt, Germany). Chloroform was purchased from Honeywell Riedel-de Haen (Seelze, Germany). Absolute ethanol was purchased from VWR International (VWR International GmbH, Darmstadt, Germany). Rat-tail collagen type I (dissolved in 0.02 N acetic acid, pH 3.3) was purchased from Corning (Corning, New York, NY, USA). Dulbecco’s Modified Eagle medium nutrient mixture (DMEM/F12), penicillin/streptomycin, Dulbecco’s phosphate-buffered saline (PBS), trypsin-EDTA 0.5% and trypan blue staining were purchased from Gibco (Grand Island, NE, USA). Ethidium D-III and calcein-AM were purchased as part of a live/dead cell staining II kit (PromoCell, Heidelberg, Germany). MG-63 (ATCC-CRL 1427) cells were purchased from ATCC. The cell proliferation reagent WST-I was purchased from Roche Diagnostics (Basel, Switzerland).

### 2.2. Scaffold Manufacturing

The PCL scaffolds were fabricated using a 3D Bioplotter made by Envisiontec (Envisiontec GmbH, Gladbeck, Germany). For this purpose, the PCL granulate was previously dried for 24 h in a desiccator at a low vacuum of 750 mbar. It was then transferred to the supply cartridge of a high-temperature viscous dispensing head for the 3D Bioplotter. Prior to the printing process, the PCL in the cartridge was heated to 80 °C. Subsequently, 3D printing was performed at a speed of 1 mm/s and 4 bar of pressure on a Plexiglas plate. A 24G needle from Envisiontec with an inner diameter of 550 µm was used for printing. The Plexiglas plate on which the print was made was cooled down to 11 °C by means of a piezo element. Each scaffold was printed in 12 layers. In order to print different outer and inner structures, certain printing parameters had to be adapted to the respective pattern. These were the needle offset, the pre-flow, and the post-flow. The needle offset specifies the distance between the base and the printer needle. This parameter was important because the appearance of the first layer affects the other layers. The pre- and post-flow determine how early or for how long the PCL was pushed out of the printer needle. Table 1 shows the general and Table 2 the specific parameters for the 3D printing.

#### 2.2.1. Variation of the Strand Spacing

After determining the parameters to obtain a continuous and reproducible strand, the internal structure of the scaffolds was modified. For this purpose, the 3D Bioplotter™ software from Envisiontec was used. First, straight strands were printed, which were rotated by 90° after each layer. This created a grid structure. Within this grid structure, the strand distances were then varied. First, strand spacings of 200 µm were printed, which also corresponded to a pore size of 200 µm. These spacings were then increased in 100 µm increments until the scaffolds were no longer reproducible. With the grid pattern, ribbon spacing was printed from 200 µm–600 µm. The pore size was also adjusted in the course of production from 200 µm–600 µm.

#### 2.2.2. Variation of the Outer Geometry

Various external shapes were designed using the CATIA V5-6R2019 (Dassault Systemes, Vélizy-Villacoublay, France) computer-aided design (CAD) program. The influence of the outer shape on strength should also be taken into account. Four different outer shapes were designed: a quadrilateral, a hexagon, an octagon, and a circle. By inserting the different outer shapes into Envisiontec’s 3D Bioplotter™ Software, the outer shapes could be combined with the inner structures. The hexagon with a lattice structure, the octagon with a lattice structure, and the circle with a lattice structure are shown below. In order to achieve better comparability, a strand spacing of 300 µm was chosen for the inner structure for all 4 outer shapes.

#### 2.2.3. Variation of the Inner Geometry

CATIA 5 was then used to vary the internal structure of the scaffolds. By inserting the design into the 3D Bioplotter™ software from Envisiontec, the internal structures could then be changed. This method was used to check the influence of the inner structures on the stability of the scaffolds. Three different inner structures were compared with each other. The lattice structure was compared with a wave structure and with a honeycomb structure. In order to be able to compare the inner structures better, the square shape was first chosen as the outer shape. The special feature of the honeycomb structure was that the different layers could not be rotated by 90°. Therefore, the 12 layers had to be printed in the same direction to obtain reproducible scaffolds.

#### 2.2.4. Variation of Outer and Inner Geometry

Based on the square initial shape with lines, as well as a rotationally symmetrical arrangement of the strands with an angle of 90° per layer, the variation of the inner and outer geometry was carried out in the last investigation section. For this purpose, separate round structures were filled with the 3 different inner structures (line, wave, honeycomb).

### 2.3. Characterization of the 3D-Printed PCL Scaffolds

#### 2.3.1. Characterization of Dimensions and Weight

The dimensions were measured using an electronic caliper, Precise PS 7215 (Burg-Wächter; Wetter-Volmarstein, Germany). Likewise, the scaffolds’ weight was determined using a Practum^®^ analytical balance (Sartorius Lab Instruments GmbH & CO. KG., Goettingen, Germany). Ten samples from each scaffold produced were used for this analysis. Other parameters that were determined were the strand width and pore size of the scaffolds. Images of 3 scaffolds (with at least 5 locations per scaffold and, for the wave structures, at least 9 different locations per scaffold) were generated for each mold using the VKX-210 3D laser microscope (KEYENCE Corporation, Osaka, Japan). All images were taken using a Nikon lens (Nikon Inc., Minato, Japan) with a magnification of 20× (equivalent to 400× magnification in the microscope).

#### 2.3.2. Characterization of Surface Roughness

The VK-X210 3D laser microscope from KEYENCE was also used to determine the surface roughness. All images were taken with a Nikon lens with a 50× magnification (1000× magnification in the microscope). Surface roughness was determined with the uncoated as well as the coated scaffolds (see Section 2.5). For the uncoated constructs, 3 scaffolds for each structure were measured at 3 different locations, with five measurement points each. With the collagen type-I coated scaffolds, only a single scaffold from each structure was measured at 3 different locations, with 5 measuring points each. This served only as a reference. The surface roughness was evaluated with the KEYENCE VK analysis module V3.5.0.0.

#### 2.3.3. Characterization of the Microstructure by Means of ESEM

To investigate the microstructure of the PCL scaffolds, images were generated using an ESEM FEI Quanta 250 FEG (FEI, Hillsboro, OR, USA). For this purpose, the collagen-coated constructs were stored in the FreeZone^®^ 2.5 Plus freeze-dryer (LABCONCO, Kansas City, MO, USA) over one night, then cryo-broken, and finally clamped in a sample pin holder. Thus, with an excitation voltage of 10–20 kV, the microstructure could now be recorded. In addition to the microstructure, the thickness of the collagen coating was also determined by means of the ESEM images.

#### 2.3.4. Measurement of Porosity

Porosity was determined using the images taken as reported in Section 2.3.2. The images were transferred to ImageJ (FIJI modification version 1.52 h) and the total number of pixels was determined. Then, all pores were sketched on a WACOM Cintiq Pro 24 (Wacom, Toyonodai, Japan) and their area was determined. The porosity of the samples was calculated based on the ratio between the pore area and the total area.

### 2.4. Mechanical Testing

Compressive strength was determined using the Zwick/Roell Z005 (Zwick/Roell, Ulm, Germany) universal testing machine. After the samples were placed in the universal testing machine, the thickness was determined with a maximum load of 1 N. Once the surface was reached, the actual measurement took place. The termination criteria for the compression test were 2000 N and deformation of 50%. The measurements were performed according to the standard DIN EN ISO 604: 2019, with at least 5 different samples per geometry variation.

### 2.5. Coating with Collagen-I

The collagen coating of the PCL scaffolds was performed as described elsewhere: after the scaffolds were prepared, they were first placed in a 24-cell culture plate (well plate) and cleaned with an ascending ethanol series (30%, 50%, 70%, 80%, 96%, 100%) five times for each alcohol series. During the 30-minute drying time under a fume hood, the collagen was prepared. For this purpose, 600 µL of collagen type I ([c] = 3.77 mg/mL; dissolved in 0.02 N acetic acid, pH 3.3) was added to the individual wells of a new 24-well plate. After drying, the surface of the scaffolds was modified with a Piezobrush^®^ PZ3 (Relyon Plasma GmbH, Regensburg, Germany) for 1 minute, with a 10-second break after 30 seconds. The short break ensures that the PCL is not melted by the Piezobrush^®^ PZ3. It is also important to move the Piezobrush^®^ PZ3 evenly. The subsequent insertion of the scaffolds into the collagen had to be performed quickly in order not to destroy the effect of the previously performed treatment. The scaffolds, now placed in collagen, were then stored for 72 h on the rocker shaker (IKA^®^ Rocker 2D Basic, Staufen, Germany) in the refrigerator at 5 °C so that the scaffold could be completely enclosed by the collagen. At the end of 72 h, the scaffolds were transferred to a new 24-well plate and dried for 24 h in the Memmert IN incubator (Memmert, Schwabach, Germany) at 37 °C. Finally, the collagen layer on the scaffolds had to be treated in such a way that the individual collagen molecules form a better connection/crosslinking with each other. Thus, after the drying process, the scaffolds were provided with a crosslinking solution. For this purpose, 1 mL of EDC solution (1 mL 95% ethanol, containing 9.585 mg EDC powder) was added to each scaffold and incubated for 16 h at room temperature. Before the biocompatibility tests could be performed, the coated scaffolds had to be rinsed and cleaned again (5 times with distilled water and 3 times with 70% ethanol).

### 2.6. Biocompatibility

MG-63 cells (ATCC, CRL 1427) were used for all biocompatibility tests. All tests were performed with 25,000 cells/100 µL per scaffold. Per test, 10 identical scaffolds per geometry variation were used and all tests were repeated at least 3 times. The biocompatibility tests were carried out exclusively with the mechanically most stable scaffold types.

#### 2.6.1. Live/Dead Assay

On each scaffold, 100 µL of the medium were pipetted with 25,000 cells/100 µL of MG-63. The well plates were then incubated for 2 h at 37 °C and at a CO_2_ saturation of 5% in an incubator. After two hours, 1 mL of the specific cell medium described previously was added to each well before incubating the well plates in the incubator for 3, 7, and 10 days. The staining solution was prepared by adding 2 mL DPBS (art. no. 14190-094, Gibco, Grand Island, NE, USA) to a Falcon tube (Greiner Bio-One International GmbH, Kremsmünster, Austria) and 4 µL ethidium homodimer III (Eth D-III) solution (together with the calcein part of the Live/Dead Cell Staining Kit II (PromoCell, Heidelberg, Germany)), according to the manufacturer’s protocol (PromoCell). Then, 1 µL of calcein dye was added after mixing the staining solution. All steps were performed in the dark to avoid photobleaching of the staining solution and samples. To eliminate serum esterase activity, all samples at a specific time point had the medium removed and the cells washed. Staining was then performed according to a previously published protocol [34]. The evaluation was performed using an Olympus fluorescence microscope (BX51, Olympus, Osaka, Japan) at 5 different positions on the samples, at 5× and 10× magnification.

#### 2.6.2. Cell Proliferation (WST-I)

Cells were again seeded on the scaffolds and, as a control, on Thermanox coverslips in the same number and concentration as in the previous biocompatibility tests. After two hours of incubation in the incubator at 37 °C and 5% CO_2_ saturation, the cells adhered to the scaffolds and Thermanox coverslips (as a control) so that the respective medium (1 mL) could be added. Plates were then incubated in the incubator for 3, 7, and 10 days. For this purpose, all medium was aspirated, and all wells were washed three times with PBS. Then, the scaffolds and Thermanox coverslips were transferred to a new 24-well plate. In the old well plate, 300 µL of DMEM medium without phenol red (additives: 1% FBP and 1% P/S) was added to each of the wells where the scaffolds and membranes had been previously. In the new well plate, 600 µL of the same medium was added to each of the wells containing the scaffolds and membranes. The blank contained only DMEM medium without phenol red (with the same additives) and was measured to account for background absorbance. Finally, 10% WST was added to all samples of the respective measurement time point (3, 7, 10 d) and incubated for 2 h in the incubator. After 2 h, the absorbance was measured using a spectrometer at 450 nm.

#### 2.6.3. Cytotoxicity (LDH Assay)

LDH measurements were performed after 24, 48, and 72 h. In addition to the scaffolds, positive controls (Triton X, 100% toxicity) and negative controls (cells only, 0% toxicity) were used for the measurements at different times. Both coated and uncoated scaffolds were also used. Cells were seeded onto the scaffolds and membranes in 100 µL of their medium (MG-63: 25,000 cells/100 µL). These were then incubated for 2 h in an incubator at 37 °C and 5% CO_2_ saturation. Following this, 1 mL of DMEM-F12 medium without phenol red was added to all wells, with the additions of 1% P/S and 1% FBS. Since FBS in higher concentrations may induce background absorption, only 1% FBS was used. In the positive controls (C+), an additional 1% Triton X 100 was added to kill 100% of the cells. After 24 h incubation in the incubator, 100 µL from each well was transferred to three new wells of a 96-well plate. Thus, from 1 well, 3 wells of 100 µL each were obtained. To ensure that the “blank” had the same concentration of phenol red, 100 µL of DMEM-F12 medium containing phenol red was added to this well, prior to transfer. To evaluate the cytotoxicity, the Cytotoxicity Detection Kit solution was prepared. For this, 111.1 µL of catalyst solution was mixed with 5 mL of staining solution. Of this, 100 µL was pipetted into each well before incubating the well plate in darkness for 30 min. At the end of the 30-minute period, the absorbance at 490 nm could be measured using a spectrometer. The experiment was performed a total of 4 times.

### 2.7. Statistics

All values in this paper are expressed as mean ± standard deviation. The associated calculations were performed using an Origin 2020 Professional SR1. An ANOVA (Tukey test) was used for significance testing (*p* < 0.05).

## 3. Results

### 3.1. Characterization of the 3D Printed Scaffolds

#### 3.1.1. Characterization Dimensions and Weight

**Figure 1:**
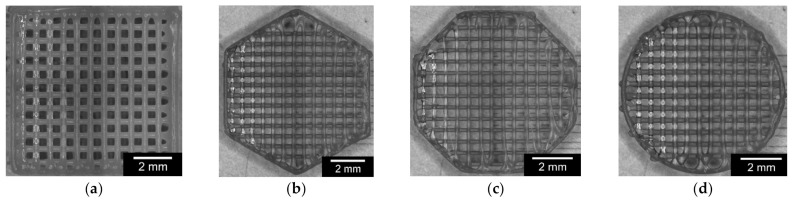
Comparison of PCL scaffolds with variations in outer geometry: (**a**): square; (**b**): hexagon; (**c**): octagon; (**d**): circle.

The PCL scaffolds, with variations of the outer geometry, showed a relatively similar value in length/diameter, ranging from 8.04 + 0.07 mm for the circular outer geometry to 8.72 + 0.05 mm for the square geometry. Within each geometry, the printouts were very constant, with standard deviations ranging from 0.03 to 0.07 mm. The height of the different scaffolds, which always consisted of 12 layers, was more varied. The octagon had the lowest value of 2.14 + 0.09 mm and the square had the highest value of 2.85 + 0.04 mm. Pore size and strand width were identical for all samples, with 300 µm strand width and 295.5 µm pore size. For the sake of comparability, only complete pores/strands were considered. Figure 1 shows the PCL scaffolds (image taken by the 3D Bioplotter after completion of the printing process) and all values assigned to the individual geometries in Table 3.

**Figure 2:**
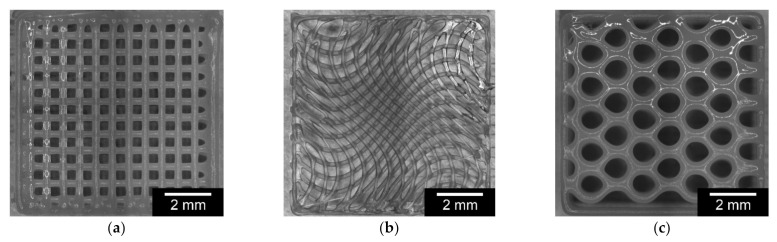
Comparison of square PCL scaffolds with different inner structures: (**a**): line; (**b**): wave; (**c**): honeycomb.

In the variation of the inner geometry based on the square scaffolds, the honeycomb structure showed the highest weight at 139 mg. The scaffolds with a line structure weighed the least, at 120 ± 20 mg. Due to the fact that all scaffolds were based on the same outer geometry, the length of 8.71 mm and height of 2.84 mm did not differ significantly. An overview of the variation of the internal geometry is shown in Figure 2, with images taken by the 3D Bioplotter directly after finishing the 3D printing process. Table 4 summarizes all results of the variations in the inner geometries. Looking at the strand widths, it becomes clear that these differ significantly, due to the geometry. In particular, the honeycomb structure has thicker strand widths of 340 µm. The pore size is also larger than in the line structure, due to the geometry. The wave structure shows similar strand thicknesses as the line structure, but the pores are very different. In the core area of the scaffold, the pores are quite uniform and become increasingly larger toward the edge (see Figure 2).

**Figure 3:**
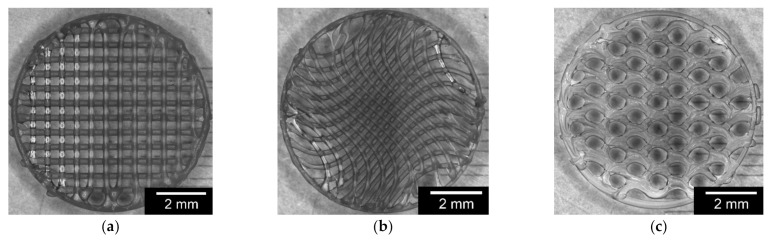
Comparison of round PCL scaffolds with variations in inner geometry: (**a**): lines; (**b**): waves; (**c**): honeycomb.

The variation of outer and inner geometry in terms of round scaffolds is shown in Figure 3. The round scaffolds with a line inlay showed the lowest weight of 44.71 mg, whereas the scaffolds with a honeycomb inlay (57.74 mg) have a 29% higher weight. Since all scaffolds are based on the same CATIA V5 file, the diameters and heights of the different scaffolds do not differ significantly. However, the pore sizes and strand thicknesses showed significant differences. The wave structure had the smallest pores at 244 µm and the honeycomb structure had the largest pores at 300 µm. The strand widths did not vary as much and were in the same range of between 258 and 267 µm for the wavy and line structures and 294 µm for the honeycomb structure. Figure 3 shows images of the 3D Bioplotter after completion of the printing process. Table 5 summarizes the results of the variation inner/outer geometry (related to the square scaffolds).

#### 3.1.2. Characterization of Surface Roughness

**Figure 4:**
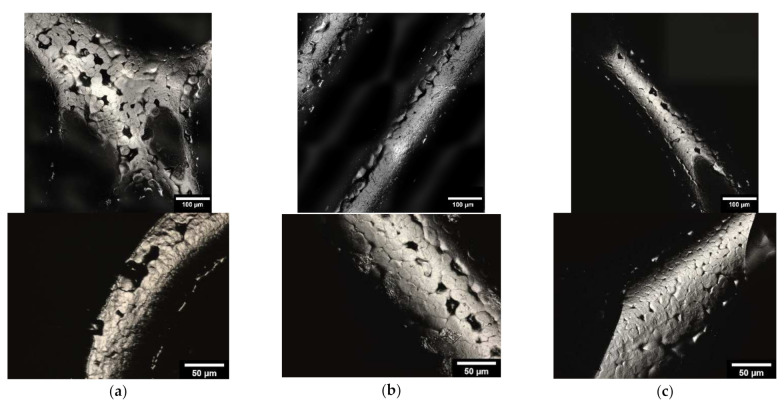
Comparison of the surface roughness for uncoated (**top**) and coated (**bottom**) PCL scaffolds, with variations in the infill structure (**a**): honeycomb; (**b**): wave; (**c**): line structure. Images were taken with a KEYENCE VK-X210 3D laser scanning microscope.

The surface roughness was measured with the uncoated and coated scaffolds (see Figure 4). For this purpose, 15 uncoated and 15 coated test samples were averaged for the three shapes, the details of which are listed in Table 6. A difference between the two groups could be noted. For example, the wave and line shapes with the coated scaffolds exhibited less roughness than the plain constructs. There are no significant differences between the individual values for the uncoated and coated samples. On average, the surface roughness of the uncoated samples was 5.42 ± 0.82 µm, with 2.75 ± 0.48 µm for the coated sample. This difference is considered significant.

#### 3.1.3. Microstructure Analysis by Means of ESEM

**Figure 5:**
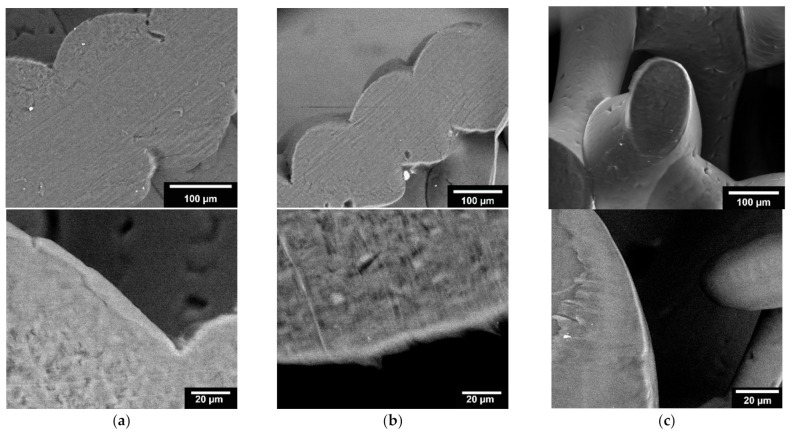
ESEM image of PCL scaffold: (**a**): honeycomb structure; (**b**): wave structure; (**c**): line structure; top: 800×; bottom: 2500× magnification. Images were taken with an FEI Quanta 250 FEG, backscattered; acceleration voltage 10 kV.

Microstructure analysis with ESEM allowed the coating to be examined in more detail. Figure 5 shows example images of the three different infill shapes (honeycomb, wave, and line). Additionally, the thickness of the collagen was determined for the different shapes. The waveform had the thickest coating, with a collagen layer of 5.57 ± 1.46 µm. The other two shapes, on the other hand, were slightly less thickly coated but were very close to each other (honeycomb shape: 4.50 ± 0.69 µm; line shape: 4.86 ± 0.46 µm).

### 3.2. Mechanical Testing

#### 3.2.1. Variation of Strand Spacing

**Figure 6:**
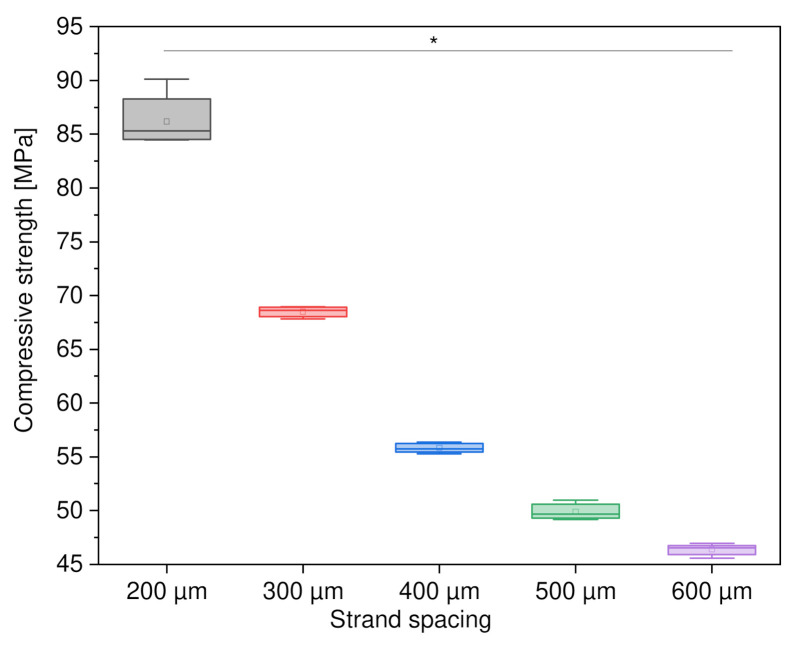
Compressive strength as a function of strand spacing; *n* = 30; *p* < 0.05 (*).

First, the variation of the strand spacing with square PCL scaffolds was investigated. It was found that the scaffolds with a strand spacing of 200 µm were the most stable, at 86.18 ± 2.34 MPa. At 300 µm strand spacing, the compressive strength decreased to 68.49 ± 0.47 MPa. The scaffolds with 600 µm strand spacing were the weakest, with a compressive strength of 46.38 ± 0.52 MPa. Figure 6 shows a comparison of compressive strength as a function of strand spacing.

#### 3.2.2. Variation of Outer Geometry

**Figure 7:**
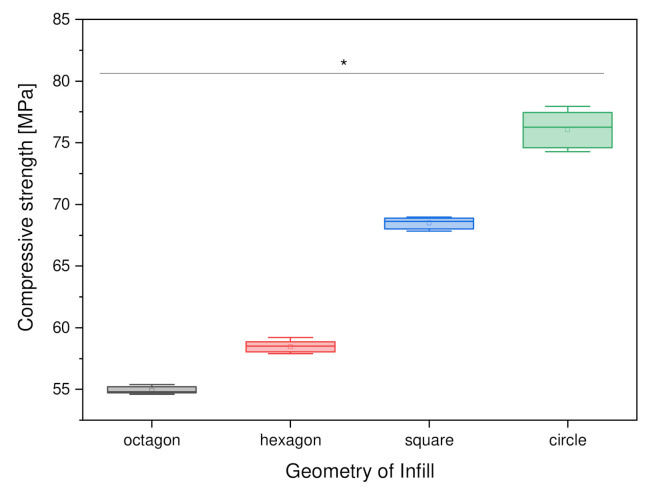
Compressive strength as a function of the outer geometry; *n* = 30; *p* < 0.05 (*).

The comparison of the compressive strength as a function of the external geometry yielded values in a range from 52 to 76 MPa. The samples with octagonal external geometry showed the lowest compressive strength, with a value of 52.96 ± 0.98 MPa, whereas the circular samples demonstrated the highest compressive strength at 76.07 ± 1.49 MPa. Figure 7, below, shows the comparison of the compressive strength of the PCL scaffolds as a function of the external geometry.

#### 3.2.3. Variation of Inner Geometry

**Figure 8:**
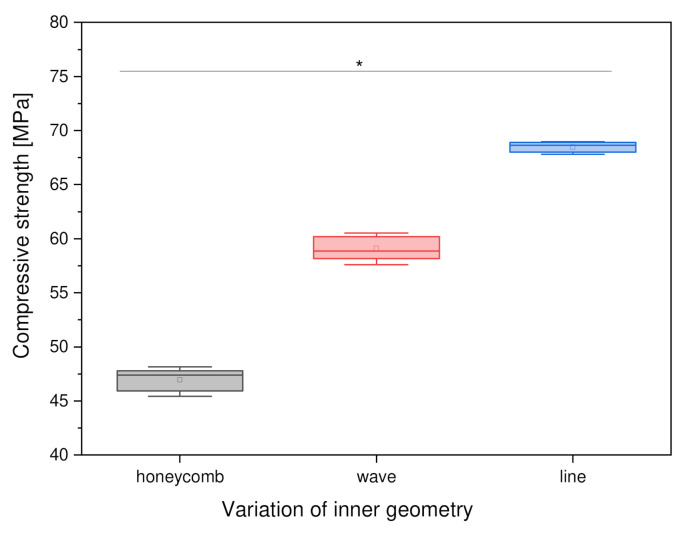
Compressive strength as a function of the inner geometry, *n* = 30; *p* < 0.05 (*).

When investigating the influence of the internal structure on the compressive strength of square PCL scaffolds, the honeycomb structure was found to have the lowest compressive strength, with a value of 46.96 ± 1.07 MPa. The scaffolds with a grid pattern had the highest compressive strength of 68.49 ± 0.47 MPa. The scaffolds with a wavy infill were 59.11 ± 1.13 MPa. Figure 8 shows a comparative bar chart.

#### 3.2.4. Variation of Outer and Inner Geometry

**Figure 9:**
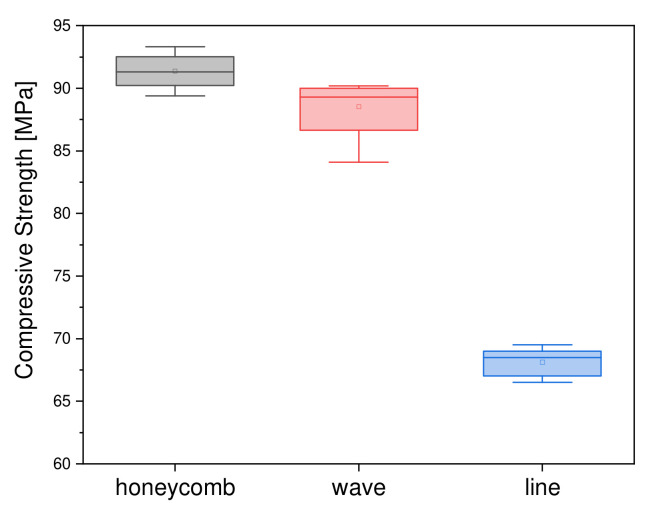
Boxplot of compressive strength as a function of outer and inner geometry (round samples).

For the variation of the inner and outer geometry, the geometry was selected that achieved the highest values with regard to compressive strength in the measurements previously carried out: the round outer shape. During our further investigations, the infill was varied. Here, the honeycomb structure showed the highest values at 91.4 ± 1.4 MPa. The round structure with a line infill achieved the lowest value in terms of compressive strength, with a value of 68.1 ± 1.1 MPa. Figure 9 summarizes the dependence of compressive strength on the outer and inner shape.

### 3.3. Biocompatibility

#### 3.3.1. Live/Dead Assay

**Figure 10:**
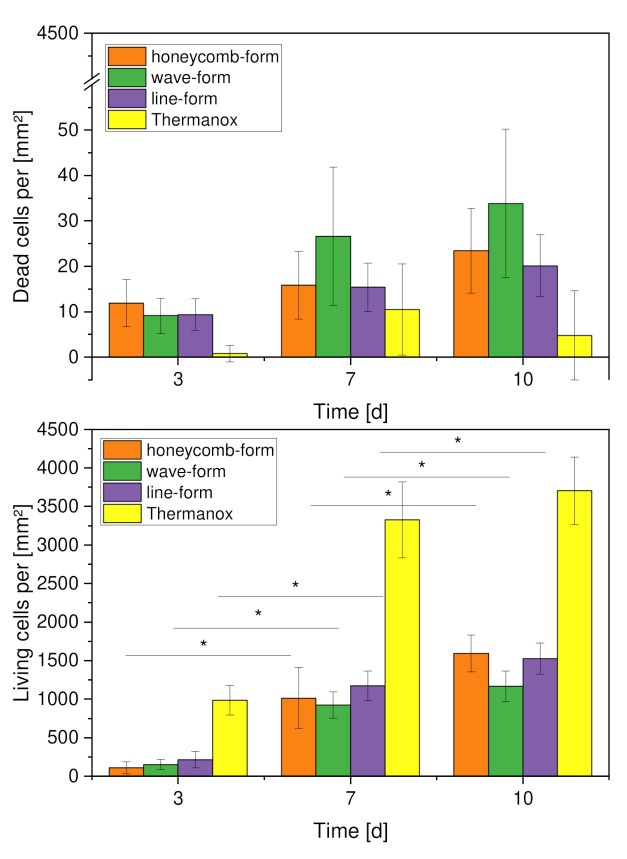
Comparison of living and dead cells per mm^2^ for the geometry variations, *n* = 30; with *p* < 0.05 (*).

During the live dead cell analysis, it was shown that there was an increase in MG-63 cells over the individual days of the experiment (days 3, 7, and 10) for both the living cells and the dead cells. For the living cells, this increase was significantly higher from day 3 to day 7 than from day 7 to day 10. For the dead cells, on the other hand, there was an even increase across the test days (see Figure 10). Additionally, the honeycomb shape had fewer live cells on day 7 (1013 ± 397) than the line shape (1172 ± 191), but this was reversed on day 10 (honeycomb shape: 1591 ± 239; line shape: 1526 ± 201). The waveform dropped significantly in terms of the number of live cells, especially on day 10 (1167 ± 197). The Thermanox coverslip, on which the MG-63 cell line showed optimal growth conditions, had at least twice the number of live cells on all experimental days (day 3: 984 ± 190; day 7: 3327 ± 492; day 10: 3704 ± 437). At the same time, very few dead cells were counted on the Thermanox coverslip (day 3: 1 ± 2; day 7: 11 ± 10; day 10: 5 ± 10). More dead cells were documented in the geometries, although this represents a small proportion relative to the living cells. There were no significant differences among the shapes on day 3. However, on day 7, a significant difference was evident between the wave and line shapes, which also occurred on day 10. Likewise, a significant difference was visible between the honeycomb and wave shapes on day 10. Looking at the individual shapes on the test days among themselves, it was found that there was a significant difference in all three shapes between day 3 and day 7. The same applied, regarding the examination of significance between day 7 and day 10, where significant differences were also found for all forms.

**Figure 11:**
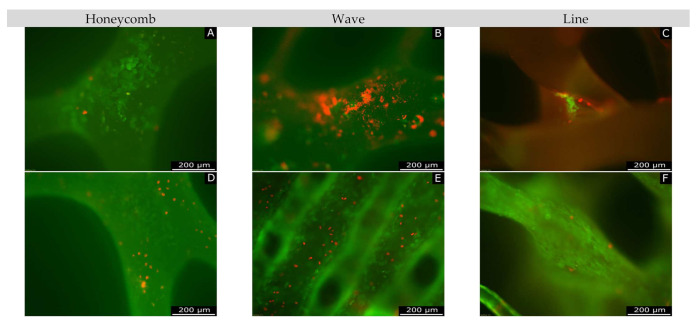

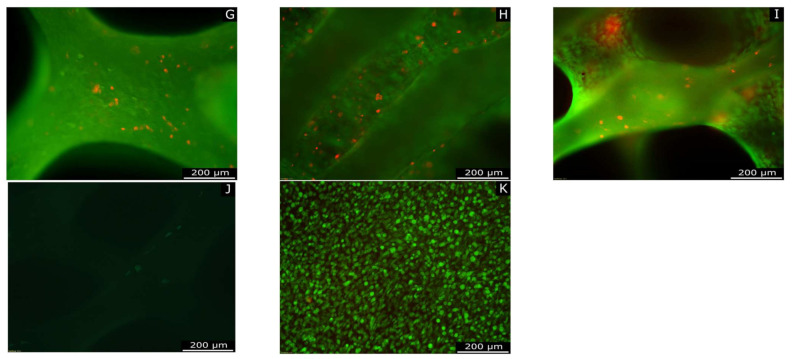
Overview of live/dead staining; (**A**–**C**): day 3; (**D**–**F**): day 7; (**G**–**I**) day 10; (**J**): intrinsic fluorescence of scaffolds; (**K**): Thermanox coverslip on day 10.

Figure 11 shows live/dead images of each shape, taken with the fluorescent microscope. Here, the live cells are shown in green and the dead cells in red. The images show a steady proliferation of the MG-63 cell line from day 3 (A to C) to day 7 (D to F), then to day 10 (G to I). This was true for both live and dead cells. The visual representation of the Thermanox coverslip at day 10 (K) illustrates that the cells proliferated much better on the Thermanox coverslip, where virtually no dead cells were present. Furthermore, the scaffolds exhibited low to no intrinsic fluorescence (J). Additionally, it can be seen that the honeycomb and line forms were covered with more live cells on day 7 and also on day 10. The low percentage of dead cells, in all constructs, was also evident in the images.

**Figure 12:**
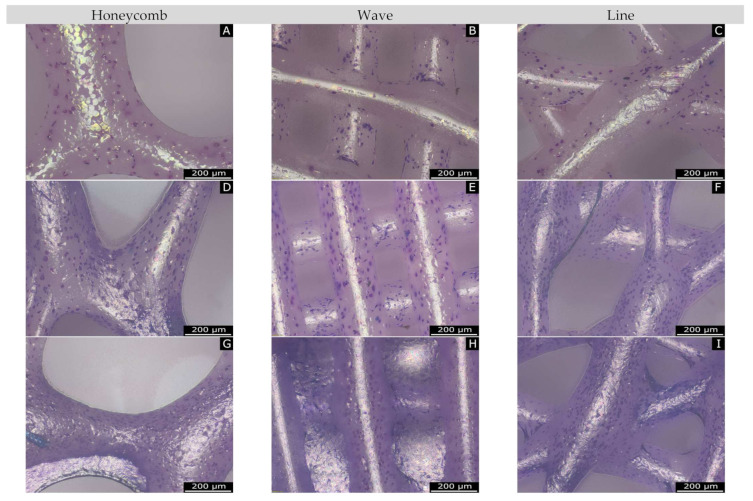
Overview of Giemsa staining. (**A**–**C**): day 3; (**D**–**F**): day 7; (**G**–**I**): day 10, for the different geometries.

The Giemsa-stained images (Figure 12), in which all cells (live and dead) are visible, made it clear that cells proliferated strongly from day 3 (A to C) to day 7 (D to F) up to day 10.

#### 3.3.2. Cell Proliferation

**Figure 13:**
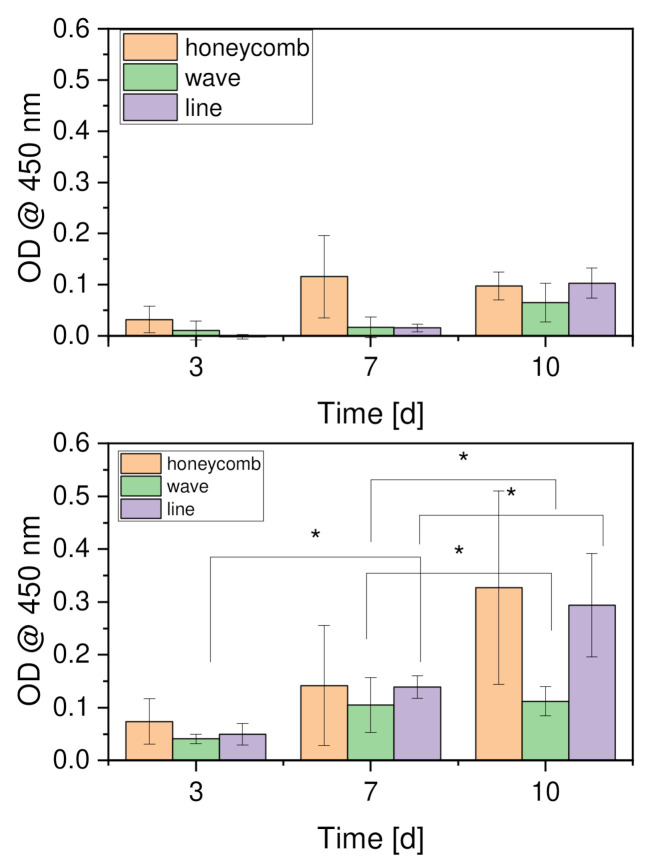
Cell proliferation assay (WST-I). Top: results of the remaining cells in the cell culture plate; bottom: results of the cells on the scaffolds; *n* = 30; with *p* < 0.05 (*).

A WST-I assay was performed to test cell proliferation. The scaffolds were transferred to a new cell culture plate before each measurement. In addition, the remaining cells in the original cell culture plates were also examined. Figure 13 (top) shows the proliferation of the remaining cells and Figure 13 (bottom) shows the proliferation of the cells on the scaffolds. An increase in proliferation can be seen over the 10-day period. Compared with one another, the cells on the honeycomb structure showed the highest proliferation, which increased up to day 10. The same applied to the line structure, with slightly lower values. However, there were no significant differences between the honeycomb and line structures. The wave structure showed the lowest proliferation in comparison to day 10. Furthermore, it can be seen in Figure 13 (bottom) that this growth stagnated after day 7.

#### 3.3.3. Cytotoxicity

**Figure 14:**
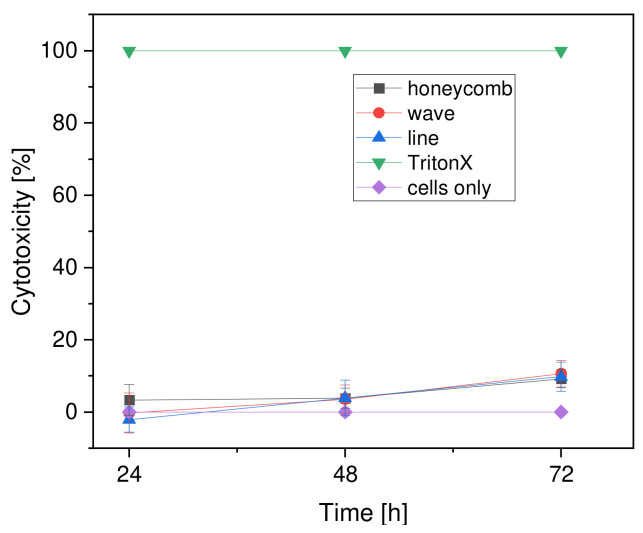
Overview of the cytotoxicity of the different geometries compared to negative (cells only) and positive (TritonX, all cells dead) controls.

No significant cytotoxicity was evident after 24 h for any of the scaffold shapes, with the wave (−0.26 ± 5.49%) and line (−2.13 ± 3.46%) shapes even showing negative values. The cytotoxicity of the honeycomb shape was 3.30 ± 4.25% (Figure 13). From 24 h to 48 h, the line shape (3.86 ± 4.92%) showed the greatest change and the honeycomb shape (3.81 ± 2.78%) showed very little change. After 72 h, the percentage of cytotoxicity in the forms ranged from 9% to 11% (honeycomb form: 9.19 ± 2.47%; wave form: 10.61 ± 3.6%; line form: 9.76 ± 3.99%). In addition, a positive control (Thermanox with Triton X) was included, in which all cells died due to the Triton X (100% cytotoxicity). The negative control (Thermanox with cells) had cytotoxicity of 0%. Overall, the values of the percentage of cytotoxicity in all scaffold forms were very close to each other and, most importantly, in a very low range. Therefore, the values were much closer to the negative control than to the positive control (see Figure 14).

## 4. Discussion

### 4.1. Characterization of the Scaffolds

No great differences were detected in diameter and height for all three shapes (line, wave, and honeycomb). This indicates that the 3D Bioplotter had no problems, firstly with the circular outer structure, and secondly, with the number of layers. On the other hand, it was noticeable that the line shape had significantly less weight (about 10 mg) than the honeycomb and wave shapes. One reason for this could be the twisting of the individual layers since less material may have been extruded from the needle. In general, the TE must be able to fabricate porous 3D scaffolds, and square 3D scaffolds are often printed [32]. Woodfield et al. [35] also printed square rectilinear layers with a 90° rotation. Thus, circular printing with different internal structures is rarer. Roopavath et al. [36] described the variation of the percentage infill of square specimens. More material was printed, which reduced the number of pores and halved sintering-induced shrinkage while increasing the compressive strength by a factor of 6. Sears et al. [37] described the fabrication of scaffolds from various printable materials, such as PCL and high internal phase emulsions (HIPE). They also relied on round outer geometries with linear infill. The outer round structure was printed from PCL or PLA, while the linear infill was made from HIPE emulsion ink. In addition, they varied the proportion of infill from 70 to 100% and achieved printing strengths of up to 10 MPa. Soufivand et al. [38] printed different infills consisting of waves or oblique lines. However, they only discussed the influence of different filaments on printing success. Mechanical testing of the 3D printed scaffolds was not performed. Cea et al. [39] described a parameter study on PLA and PCL printed scaffolds for use with cartilage tissue. They printed different types of pores consisting of square-, triangular- and ellipsoidal-shaped pores in PLA and PCL and studied the printing success; similar to the present study, they concluded that further studies should be conducted to investigate the influence on cell colonization. Variations between the different internal structures were also found in the pore size and strand width measurements. The pore size was set to 250 µm and the strand width to 300 µm by default. For the honeycomb shape, the strand width was close to the default size, with the pores appearing much larger. In addition, as seen in Figure 3, the pore shape was printed in a circular shape rather than in a hexagonal one, as intended. It is possible that the 3D printer had a problem with the hexagonal structure and therefore could not accurately reconstruct the shape. The situation was different for the other two shapes. On the one hand, the values of the strand width of the wave and line shapes were far from the set parameter of 300 µm; on the other hand, the pore sizes were within the specified size. Normally, the thickness of the strand width affects the pore size. Therefore, the pore sizes of the wave and line shapes should be larger than those of the honeycomb shape. However, this was not the case. Reasons for this could be that in the wave and line form, the twisting of the individual layers could play a role. It could also be that the twists caused the pore sizes to be smaller and, therefore, within the specified range. Likewise, the twists could be responsible for the small strand width. Due to the twists of the layers, the individual strands of a layer were never extruded exactly on top of one another and thus experienced lower pressure in the upper layer, which conclusively means that the strands were not pressed apart. The large standard deviations of all inner geometries were very noticeable, both in pore size and strand width. In the case of the waveform, for example, this variation is readily apparent in Figure 3. Here, the mean pore sizes were significantly smaller than the pore sizes at the edge of the scaffolds. These high standard deviations also indicate that the outer circular structure and the additional inner geometries with the individual twists of the layers presented a great challenge for the 3D Bioplotter. In particular, the precision and accuracy of the printing may have suffered. In general, these deviations between the individual parameters and between the different inner geometries may have other reasons. One reason could be the ambient temperature of the printer, which was not always the same. Furthermore, a minimal unevenness of the platform on which the PCL was extruded is sufficient to cause relatively large changes in the dimensions when working at this order of magnitude. The amount of PCL placed in the cartridge could be another factor. Thus, the question arises of whether the PCL was always under the same conditions when printing began.

### 4.2. Surface Roughness

When comparing the surface roughness of the uncoated and collagen type-I coated scaffolds, a difference can be seen. It would be expected that the surface roughness of the coated scaffolds would be lower since the collagen should distribute evenly on the scaffolds and form a uniform layer. This was not the case with the honeycomb shape, unlike the wave and line shapes. The honeycomb shapes that were coated with collagen had a higher surface roughness, which could well be due to the larger pores and to the non-existent twisting of the layers. It is possible that this is why the collagen did not distribute well or evenly. Glowacki et al. [40] fabricated whole scaffolds from collagen but did not measure the surface roughness. Kim and Kim [41] achieved similar surface roughness values of 2.4 ± 0.2 µm with their self-healing collagen coatings, after treatment with water. In our previous work, we showed that the uncoated scaffolds had a surface roughness of 4.11 ± 0.27 µm and the collagen-coated samples had a roughness of 3.35 ± 0.3 µm [34]. The scaffolds were square, with a line infill. Compared with the values for the roughness of the scaffolds with line infill, the values are slightly different, which may be due to the fact that a new batch of PCL and collagen-I was used for the present work. In addition to the collagen coating of the PCL scaffolds, the PCL scaffolds were also often coated with hydroxyapatite. This procedure has been followed, for example, by Beşkardeş et al. [42]. However, the surface roughness of the pure PCL constructs and that of the hydroxyapatite-coated constructs was not measured here either. In contrast, in one of our other studies, the surface roughness of inverse β-TCP scaffolds was determined; these were between 7 µm and 10 µm at different strand widths, thus exhibiting higher values than the pure and coated PCL scaffolds of this work [43].

### 4.3. Microstructure by Means of ESEM

With the use of ESEM images, it can be clarified that a collagen layer is present. Kim and Kim [41] also used the ESEM for their work to determine the thickness of their self-healing collagen layers. The thickness of the collagen layer in our scaffolds was lowest in the honeycomb shape, which could be an indication that the collagen will deposit or adhere most poorly in this shape. The less the amount of collagen that was deposited onto the scaffolds, the rougher the surface.

### 4.4. Compressive Strength

#### 4.4.1. Variations of Strand Spacing

For the compressive strengths of the quadrilateral shape with different strand spacing of 200 µm–600 µm, it was observed that the compressive strength is highest at 200 µm at 86.18 ± 2.34 N/mm^2^. The lowest compressive strength had a strand spacing of 600 µm with 46.38 ± 0.52 N/mm^2^. This is related to the fact that with a strand spacing of 200 µm, the porosity was smaller, and more PCL was used compared to shapes with strand spacings of 300 µm–600 µm. This was also shown by the other compressive strengths between 300 µm and 600 µm. The larger the strand spacing, the smaller the compressive strength of the scaffolds. In addition, the loaded area became smaller according to strand spacing. However, it was noticeable that the difference between the compressive strengths decreased more and more. The difference (of compressive strength) between 200 µm and 300 µm was still 17.69 N/mm^2^. This was the highest value at distances of 100 µm. For the strand spacing of 500 µm–600 µm, the difference was only 3.51 N/mm^2^. This represented the lowest value, between strand spacings. This could be related to the fact that the outer structure of the scaffolds was always the same. Another point could be that the porosity does not increase as much when the strands are spaced further and further apart. Bittner et al. [44] also studied PCL scaffolds with different pores and found in their work that small strand spacings or pore sizes had a higher compressive modulus. This is in agreement with the compressive strength found for the different strand spacings in this work, as well as in the previously published work by our group, which was in connection with inverse 3D printing with variations in the strand width of the resulting scaffolds for use as a bone replacement [45]. Götz et al. [46] also described the decrease in compressive strength with increasing strand spacing. In their work, however, they investigated scaffolds made of calcium magnesium phosphate cement.

#### 4.4.2. Variation of the Outer Geometry

Among the outer shapes, the circular shape had the highest dynamic compressive strength at 76.07 ± 1.49 N/mm^2^. The octagon shape had the lowest dynamic compressive strength at 52.96 ± 0.98 N/mm^2^. In general, it can be seen that the more corners the external shape had, the lower the dynamic compressive strength. Thus, the square shape had a compressive strength of 68.49 ± 0.57 N/mm^2^, while the hexagon shape had a strength of 57.31 ± 1.65 N/mm^2^. This may be due to the influence of porosity on the compressive strength. Thus, the circle had the lowest porosity at 26.4% and the highest compressive strength at 76.1 ± 1.5 MPa. In contrast, the octagonal structure had the highest porosity at 34.9% and the lowest compressive strength at 54.9 ± 0.3 MPa. However, the square with the second-highest value for porosity (31.9%) also shows the second-highest value of 68.5 ± 0.5 MPa in compressive strength. This may be related to the fact that the printer has difficulty depositing the PCL evenly at the corners. This can result in small gaps between the individual layers and, hence, can affect the stability of the outer forms. Since there are no corners in the circular shape, the PCL is always deposited evenly. Nevertheless, the circular shape remains the most stable, even if this problem could be solved. Regarding the change in the outer structure of PCL scaffolds, in terms of dynamic compressive strength or other stability characteristics, there are no publications on this topic, so the values could not be compared.

#### 4.4.3. Variation of the Inner Geometry

The compressive strengths of the internal structure showed that the lattice pattern had the highest compressive strength at 68.49 ± 0.47 N/mm^2^. The wave pattern came second at 59.11 ± 1.13 N/mm^2^, with the honeycomb pattern having the lowest compressive strength at 46.96 ± 1.07 N/mm^2^. The influence of porosity was apparent since the sample with the lowest porosity (i.e., the line-structured infill with 26.4% porosity) exhibited the highest compressive strength (68.49 ± 0.47 MPa), whereas the infill structure with a higher porosity of 32.2% (i.e., the wave infill) exhibited the second-highest compressive strength (59.11 ± 1.13 MPa). One reason for this finding is that the honeycomb pattern could not be rotated by 90° after each layer, which means that the loaded area was smaller than with the grid and wave patterns. Furthermore, the porosity was highest in the honeycomb pattern, since it was not possible to print smaller honeycomb structures within the parameters mentioned. The problem with the wave pattern is that the distances between the strands and the edge of the structure become larger and larger. So far, this problem cannot be solved for the wave pattern. This leads to a loss of stability in the wave pattern since the loaded area is smaller and the scaffold cannot be loaded symmetrically. Balakrishnan et al. [33] also measured different infills on round samples in their work. However, they used commercially available PLA filament as well as a filament printer. Depending on the degree of infill, there were differences in compressive strength between the different printed internal geometries (diamond, hexagon, square, triangle). In terms of our nomenclature, all the inner Balakrishnan’s [33] structures are line structures.

#### 4.4.4. Variation of the Inner and Outer Geometry

In the variation of the outer and inner structures, the strongest load-bearing structure from the previous tests was selected: the round outer structure. In contrast to the previous tests, it was found that the honeycomb had the highest load-bearing capacity. The influence of porosity showed a different effect when varying the outer and inner geometry than in the previously considered variants. Thus, the honeycomb structure with the highest porosity of 31.8% also had the highest compressive strength (91.4 ± 1.4 MPa), in contrast to the line infill sample with the lowest porosity of 26.4%, which also had the lowest compressive strength (68.1 ± 1.1 MPa). Balakrishnan et al. [33] also used round scaffolds (printed from PLA with a filament printer) with different infills. The type of geometry always remained the same, according to our definition of line structures, but they were 3D printed at different angles: 90°, 45°, and as diamond structures. In addition, the level of infill was changed. Depending on the degree of filling, the compressive strength values varied from 59.8 MPa (100% filling, regardless of geometry) to 59.9 MPa for the hexagonal infill (80%) and 36.9 MPa for a triangular infill (20% filling). Unfortunately, no standard deviations are given for the determined values, so it is not possible to establish whether there is actually a significant difference or not. Nevertheless, similar values were obtained in our tests. Overall, it was found that the round structures exhibited the highest compressive strength, regardless of infill and porosity.

### 4.5. Biocompatibility

#### 4.5.1. Live/Dead Assay

Looking at the results of the live/dead assay, it can be seen that the MG-63 cell line proliferated over each of the experimental days, 3, 7, and 10. This was significant for the live cells of all constructs. Here, it was noticeable that the live cell proliferation was significantly higher from day 3 to day 7 than from day 7 to day 10. Sajesh et al. [47] were also able to show growth of the MG-63 cell line on their constructs. Although, on the one hand, his scaffolds consisted of a chitosan/polypyrrole-alginate mixture, on the other hand, the live/dead experiment was only evaluated at 24 h and 72 h. No rapid increase was seen in the dead cells within the experimental days, so it can be assumed that the dead cells did not first develop due to an adjustment of environmental changes. Additionally, if the distribution of dead and live cells on all geometries is considered, it can be assumed that MG-63 felt comfortable on the constructs and that the coating with collagen type I worked. Among the individual geometries, the honeycomb shape had the highest number of living cells on day 10. One reason could be that the circular pore shape allowed the cells to bridge the pores better, ensuring better cell growth. Although the wave and line shapes exhibited lower surface roughness after coating, there were significantly fewer living cells on the constructs with the wave shape and slightly fewer living cells on the line shape at day 10. This is surprising because Kieswetter et al. [48] also worked with the MG-63 cell line and found that smoother surfaces had significantly more cells than rough surfaces. However, they did not use PCL for these experiments. The pore size could also have an effect on the proliferation of cells on the constructs, although no significant difference was noticed for pore sizes between constructs having 300 µm and 800 µm pore sizes [32]. The pore sizes of the wave and line shapes are not in this range, but conclusions can be drawn from them. It can be assumed that the minimal differences between the pore sizes in this work did greatly influence the cell number.

#### 4.5.2. Cell Proliferation

Absorption increased significantly over test days 3, 7, and 10 for both the new wells and the old wells. An increase in the individual forms over the experimental days is also evident in the vast majority of cases. The increase in cell proliferation over the experimental days tested in the present study was similar to that in our previous work using the same cell line [43]. In the current work, a high and rapid increase was not only evident from day 3 to day 7 with the new well but also from day 7 to day 10. The rapid increase from day 3 to day 7 could equally be explained by the change in environment from the cell culture flask to the scaffolds. However, the increase from day 7 to day 10 was not really consistent with the results of the live/dead experiment. There, the cell number did increase from day 7 to day 10, but not to this extent. The low absorption values were reflected in the low cell count per mm^2^. In addition, the honeycomb had the highest cell proliferation on day 10, which was also consistent with the live/dead assay. The results of the live/dead assay were also identical to the values of the waveform on day 7 and day 10. The extremely low increase in cell numbers coincided with the almost non-existent increase in the values of absorbance. The very high absorbance value of the honeycomb shape on day 7 could be explained by the pore size parameter. The pore sizes were in a much higher range for the honeycomb shape than for the wave and line shapes. Thus, it can be assumed that the MG-63 cells, after adapting to the environment, migrated more easily through the construct and, thus, were able to adhere more to the bottom of the well. A similar picture emerged from our previous study using inverse β-tricalcium phosphate scaffolds [43]. Hence, constructs with thicker strand widths and smaller pore sizes led to lower absorption values being measured in the old wells. Similar observations were also made by Fink et al. [49]. They observed preferential occupancy with the increasing area of the adhesion site. This finding is consistent with the previously reported “dimensional sensing” of cells on thin, one-dimensional lines interspersed with wider rectangles [50]. Larger adhesion areas result in longer dwelling times, which is in qualitative agreement with the observation that freely moving cells exhibit lower cell motility when on a larger spreading area [51,52].

#### 4.5.3. Cytotoxicity

Cytotoxicity slightly increased for all geometries over the course of the test days, which was to be expected. It is also noticeable that all the values of the geometries were very close to each other (with no significant differences) and, in general, the values were in a very low range even after 72 h with about 10% cytotoxicity. These results were also to be expected since PCL, as a biocompatible polymer, plays a major role in the field of TE and 3D printing nowadays and, thus, should not cause any negative effects on the body and cell growth [19,53]. It should be noted that the character properties, such as the pore size, the strand width, and different internal structures of the scaffolds, as well as the thickness of the collagen coating, did not show any effect on the MG-63 cell line and cell damage or death did not depend on this factor decisively. The same procedure for the LDH assay was used in one of our previous studies [43]. There, too, the cytotoxicity of the inverse β-TCP scaffolds was investigated after 24 h, 48 h, and 72 h. The results showed the cytotoxicity of the inverse β-TCP scaffolds. The evaluation showed that the scaffolds with strand widths of 500 µm, 750 µm and 1000 µm all exhibited no cytotoxicity. Thus, it was also found there that the different strand widths had no influence on cytotoxicity.

## 5. Conclusions

In the present work, we were able to show the relationship between compressive strength and geometry in terms of PCL scaffolds. Both internal and external geometry played a key role in the compressive strength of 3D printed scaffolds. We were able to show that the round shape exhibited the highest compressive strength. The type of infill (lines, waves, or honeycomb structures), as well as the porosity (but to a lesser degree than assumed), was also decisive in terms of compressive strength. The honeycomb structure, for example, proved to be the strongest infill structure, despite or primarily because no rotation of the layers took place. The different geometries also played a key role in biocompatibility. However, only the most mechanically resilient outer geometry with the different infills, the circle, was considered in the biocompatibility tests. There, the honeycomb structure again showed the best result in terms of the highest cell number/mm, as well as the highest proliferation rate of MG-63 cells. Since all constructs were printed from the same material (PCL), the results of the cytotoxicity tests were comparable. Finally, it can be stated that the choice of geometric shape plays a significant role, especially for scaffolds that are to be used as load-bearing structures.

## Figures and Tables

**Table 1:** General parameters for 3D printing.

Needle Inner Diameter (µm)	Temperature (°C)	Pressure (bar)	Speed (mm/s)	Needle Offset (mm)	Pre-/Post-Flow (s)	Platform Temperature (°C)
550	80	6.0	1.0	0.3	0.4	11

**Table 2:** Specific parameters for 3D printing.

Parameter\Shape	Honeycomb	Wave Shape	Line Shape
Needle-Offset (mm)	0.17	0.34	0.20
Pre-Flow (s)	0.70	0.55	0.20
Post-Flow (s)	0.58–0.70	−0.25	−0.10

**Table 3:** Dimensions and weight of the PCL scaffolds with variations in outer geometry.

Parameter\Geometry	Square	Hexagon	Octagon	Circle
Weight (mg)	120 ± 20	80.81 ± 2.17	44.71 ± 1.58	72.65 ± 12.87
Length (mm)	8.72 ± 0.05	8.55 ± 0.03	8.10 ± 0.06	8.04 ± 0.07
Height (mm)	2.85 ± 0.04	2.59 ± 0.01	2.14 ± 0.09	2.37 ± 0.4
Pore size (µm)	295.36 ± 9.78	295.36 ± 9.78 *	295.36 ± 9.78 *	295.36 ± 9.78 *
Strand width (µm)	300 ± 12.56	300 ± 12.56 *	300 ± 12.56 *	300 ± 12.56 *
Porosity (%)	31.9	28.9	34.9	26.4

* full-square pores considered.

**Table 4:** Dimensions and weight of the PCL scaffolds, with variations in inner geometry (starting from square scaffolds).

Parameter\Geometry	Honeycomb	Wave	Line
Weight (mg)	139 ± 24	128 ± 26	120 ± 20
Length (mm)	8.71 ± 0.11	8.70 ± 0.08	8.72 ± 0.05
Height (mm)	2.84 ± 0.07	2.83 ± 0.09	2.85 ± 0.04
Pore size (µm)	354.54 ± 36.29	244.95 ± 78.22	295.36 ± 9.78
Strand width (µm)	336.40 ± 59.78	258.75 ± 32.59	300 ± 12.56
Porosity (%)	31.2	32.2	25

**Table 5:** Dimensions and weight of the round PCL scaffolds.

Parameter\Geometry	Honeycomb	Wave	Line
Weight (mg)	57.74 ± 4.00	54.77 ± 0.89	44.71 ± 1.58
Diameter (mm)	8.14 ± 0.11	8.06 ± 0.08	8.10 ± 0.06
Height (mm)	2.14 ± 0.07	2.19 ± 0.09	2.14 ± 0.09
Pore size (µm)	300.54 ± 36.29	244.95 ± 50.22	262.19 ± 44.53
Strand width (µm)	294.40 ± 48.61	258.75 ± 32.59	267.64 ± 45.59
Porosity (%)	31.8	29.3	26.4

**Table 6:** Comparison of surface roughness of uncoated vs coated PCL, with variations in geometry, *n* = 3.

Surface Roughness (S_a_) (µm)			
Parameter\Geometry	Honeycomb	Wave	Line
uncoated	4.52 ± 2.14	5.60 ± 2.22	6.13 ± 2.84
coated	3.31 ± 1.85	2.43 ± 1.19	2.52 ± 0.82

## Data Availability

The data presented in this study are available on request from the corresponding author.
